# Meal Pattern of Male Rats Maintained on Amino Acid Supplemented Diets: The Effect of Tryptophan, Lysine, Arginine, Proline and Threonine

**DOI:** 10.3390/nu6072509

**Published:** 2014-07-01

**Authors:** Raghad Ayaso, Hala Ghattas, Mohamad Abiad, Omar Obeid

**Affiliations:** Department of Nutrition and Food Sciences, Faculty of Agricultural and Food Sciences, American University of Beirut, P.O. Box 11-0236, Beirut 1107 2020, Lebanon; E-Mails: raghadayaso@hotmail.com (R.A.); hg15@aub.edu.lb (H.G.); ma192@aub.edu.lb (M.A.)

**Keywords:** meal pattern, lysine, tryptophan, arginine, proline, threonine, rats

## Abstract

The macronutrient composition of the diet has been shown to affect food intake, with proteins having distinct effects. The present study investigated the effect of diet supplementation with individual amino acids (tryptophan, lysine, arginine, proline and threonine) on meal pattern among male rats. Meal pattern and body weight were monitored for two weeks. Proline and threonine had minimal effects on meal pattern, while the most pronounced changes were observed in the tryptophan group. Both tryptophan and lysine decreased overall food intake, which was translated into a reduction in body weight. The reduced food intake of the tryptophan group was associated with an increase in meal size, intermeal intervals (IMI) and meal time and a decrease in meal number. The decrease in the food intake of the lysine group was associated with a reduction in both IMI and meal number, and this was accompanied by an increase in meal time. Arginine increased meal number, while decreasing IMI. Proline and threonine had a minimal effect on meal pattern. Lysine seems to increase satiety, and arginine seems to decrease it, while tryptophan seems to increase satiety and decrease satiation. Accordingly, changes in meal patterns are associated with the type of amino acid added to the diet.

## 1. Introduction

Obesity is considered a public health epidemic and is associated with co-morbidities, including cardiovascular disease, diabetes and some forms of cancer [1,2]. Obesity results mainly from an inadequate balance between food consumption, which is regulated by an interaction between physiological and environmental factors, and energy expenditure [3,4,5]. Moreover, daily food intake is the outcome of eating behaviors (meal patterns) governed by several states that determine the meal size, number, time and intermeal interval: “hunger” (the physiological signal promoting the brain to initiate food seeking), “satiation” (the processes leading to the interruption of an eating episode) and “satiety” (the non-hunger state between two meals). These states are influenced by physiological and non-physiological factors [6,7]. In addition, understanding the changes in meal pattern can be a useful tool for clinicians or nutritionist to modify the diet of individuals or a population [7].

It has been reported that the macronutrient composition of the diet can significantly alter the regulation of food intake, with protein being the most satiating [8,9,10,11,12]. According to Poppitt *et al.* [13] and Stubbs *et al.* [14], protein has both short-term and long-term satiating effects in humans. Bensaïd *et al.* [15] reported that in rats, an intra-oral protein load administered at different concentrations produced a greater inhibition of food intake than an isovolumetric and isocaloric carbohydrate load. This could be explained by several mechanisms, which were proposed to be involved at the peripheral and central levels, including the alteration in gut hormone release through the suppression of ghrelin and the elevation of PYY, CCK and GLP-1, causing a reduction in appetite and food intake [12,15,16]. In addition, protein was hypothesized to reduce satiety through its capacity to stimulate diet-induced thermogenesis, which is associated with an increase in body temperature, metabolic rate and hepatic ATP production [17]. The satiating effect of proteins has been reported to differ according to protein sources [18,19,20,21,22,23]. In lean men, the satiating effect of fish protein was reported to be higher than that of beef or chicken [18], and the satiating power of gelatin (incomplete protein) was higher than that of casein [20]. However, the difference in satiating power between various protein sources may not be translated into a variation in body weight. This is indicated by the similarity in the body weight of rats maintained on high protein whey and soy diets, where the satiety of the whey protein-based diet was higher than that of soy [23]. Although both high protein diets restricted weight gain and reduced fat accumulation, each had its distinct mechanism. While the high whey protein group showed a decrease in food intake, the soy protein subjects exhibited an increase in fat oxidation.

Although the effects of protein sources and individual and/or combined amino acid supplements on the profile of ingested and plasma amino acids have been widely studied, their impact on meal pattern is not yet fully understood. We have previously investigated the impact of certain amino acids [24,25], and the present work focuses on lysine, tryptophan, arginine, proline and threonine. Lysine ingestion was reported to increase postprandial glucose clearance [26] and to stimulate the secretion of the gut hormones, CCK and GLP-1 [27]. Tryptophan is needed for the synthesis of serotonin, a neurotransmitter known to be involved in appetite regulation. Arginine is a precursor of nitric oxide and an inducer of growth hormone release [28,29], as well as proline production. Threonine was reported to improve food intake and weight gain [30]. Moreover, it was hypothesized that the central nervous system controls food intake by detecting dietary protein content and quality through the sensing of specific circulating amino acids, such as lysine [31]. The present study aims at investigating the influence of individual amino acid-supplemented diets on the meal pattern of male rats.

## 2. Experimental Section

### 2.1. Animal Housing

Adult male Sprague-Dawley rats (Animal House, American University of Beirut, Lebanon), which are known to have a good consistency in meal pattern [32], were housed initially in individual wire-bottomed cages in a room with controlled temperature (22 ± 1 °C) and under 12:12 h light-dark cycles with lights on at 7:00 a.m. The rats were moved to feed recording equipment (Model 80350 series, Campden Instruments limited, Lafayette, IN, USA), each residing in a separate chamber. Rats were allowed a four-day adaptation period while being fed a semi-synthetic control diet *ad libitum* [33] (Table 1) with a gross energy of 18.2 kJ/g distributed as 56%, 21% and 23% from carbohydrate, protein and fat, respectively. The amino acid composition of casein (g/100 g of protein) is as follows: alanine (2.6), arginine (3.6), aspartic acid (6.5), cysteine (0.4), glutamic acid (20.8), glycine (1.8), histidine (2.6), isoleucine (4.8), leucine (8.8), lysine (7.4), methionine (2.6), phenylalanine (5), proline (11.7), serine (5.4), threonine (3.8), tryptophan (1.2), tyrosine (5.3) and valine (5.7). The study was approved by the institutional animal care and use committee (IACUC) of the American University of Beirut.

**Table 1:** Composition of the control diet.

Ingredients	g/kg Diet
Casein1	198
dl-methionine	2
Maize oil	100
Sucrose	300
Corn starch	300
Mineral mix *	40
Vitamin mix **	10
Alphacel (cellulose)	50

* Modified U.S.P. XIV Salt mix; ** AIN-76A Vitamin Mixture. Dyets Inc., Bethlehem, PA, USA.

### 2.2. Experimental Protocol

The study was divided into two experiments; each experiment included a control group in which the rats were maintained on the control diet. In the experimental groups, rats were maintained on the same control diet supplemented with 5% of the specific amino acid. This translates to about 1 g/per day or 3.0 g/kg body weight per day assuming an average daily dietary intake with a body weight of 330 g. Body weights and meal patterns were monitored over two weeks.

Experiment 1: The effect of diet supplementation with 5% tryptophan or lysine on meal pattern was investigated. Thirty rats were divided into 3 equal groups (*n* = 10): control, tryptophan and lysine group.

Experiment 2: The effect of diet supplementation with 5% arginine, proline or threonine on meal pattern was investigated. Thirty rats were divided into 4 groups: control (*n* = 6), arginine (*n* = 8), proline (*n* = 8) and threonine (*n* = 8).

### 2.3. Feeding Pattern

The feed recording machine is a microstructural feeding analysis system designed for rats (Model 80350 series, Campden Instruments limited, Lafayette, IN, USA) equipped with a computer-based data acquisition system capable of monitoring feeding behavior in rodents with high sensitivity (0.1-g resolution). The system consists of 16 individual chambers with dimensions of 285 mm × 210 mm × 200 mm (L × W × H). The cage bottom is made of 2-mm rods separated by a 7-mm gap. The chambers are well ventilated to allow for air circulation. A hopper is attached to the back of the cage where food is filled. The hopper is supported by a weighing balance, which measures food weight changes, and an infrared beam that detects the animal while feeding. Time and hopper weight are logged into the computer every time the animal begins and ends feeding. Meal patterns were recorded, and the results were collected as meal number, meal size (g), meal time (s), intermeal interval (s) and feeding rate (mg/s). A meal was characterized as the ingestion of food for at least 13 s with a quantity of at least 0.3 g [34]. Meals are considered distinct if the intermeal interval is greater than 10 min [35]. Food intake was defined as the difference in food weight over 24 h and includes any intake outside the defined meals.

### 2.4. Statistics

Data are expressed as means ± SEM of all values. Data were analyzed using the Statistical analysis package for Social Sciences (SPSS, version 16, IBM, NY, USA). Data were analyzed by one way analysis of variance (ANOVA), and specific comparisons were made using Tukey’s *post hoc* comparisons. A probability of *p* < 0.05 was considered statistically significant.

## 3. Results

### 3.1. Experiment 1

Body Weight and food efficiency (Table 2): The mean initial body weight was similar among the groups. The maintenance of rats on 5% lysine or tryptophan diet for fourteen days significantly reduced their final body weight as compared to the control group. The weight gain of the control group was about 3 times higher than those of the lysine- and tryptophan-supplemented diet groups. Similarly, the food efficiency of the control group was significantly higher than those of the lysine and tryptophan groups. The final body weight, weight gain and food efficacies were similar among the lysine and tryptophan groups.

Food intake and feeding rate (Table 3): The total food intake of the tryptophan group was significantly lower than both the control and lysine groups. However, the food intake of the lysine group was significantly lower than that of the control. The diurnal (%) intake of the lysine group was significantly lower than those of the other groups, while nocturnal (%) food intake was highest in the lysine group, followed by the control and tryptophan groups. In addition, the feeding rate (total, diurnal and nocturnal) of the lysine and tryptophan groups was similar, and this was significantly lower than that of the control group.

**Table 2:** Mean weight (g), weight gain and food efficiency of rats fed on diets supplemented with 5% lysine or tryptophan.

Groups	Control	Lysine	Tryptophan	ANOVA
	(*n* = 10)	(*n* = 10)	(*n* = 10)	*p*-value
**Initial body weight (g)**	280.08 ± 10.51	285.57 ± 8.45	286.70 ± 9.313	NS
**Final body weight (g)**	392.47 ± 8.25 ^a^	323.06 ± 9.74 ^b^	319.13 ± 8.01 ^b^	<0.001
**Weight gain per day (g)**	7.22 ± 0.86 ^a^	2.50 ± 0.54 ^b^	2.16 ± 0.37 ^b^	<0.001
**Food efficiency (weight gain (g)/100 kJ)**	1.76 ± 0.165 ^a^	0.714 ± 0.11 ^b^	0.714 ± 0.11 ^b^	<0.001

^a^^,b^ Values in the same row with varying superscripts are significantly different based on Tukey’s *post hoc* comparisons (*p* < 0.05).

**Table 3:** Food intake and feeding rate of rats fed on diets supplemented with 5% lysine or tryptophan.

Groups		Control	Lysine	Tryptophan	ANOVA
		(*n* = 10)	(*n* = 10)	(*n* = 10)	*p*-value
**Food Intake**	Total (kJ/day)	403.85 ± 9.83 ^a^	338.7 ± 11.1 ^b^	295.75 ± 7.83 ^c^	<0.001
	Nocturnal (%)	71.94 ± 1.36 ^a^	79.51 ± 1.09 ^b^	67.99 ± 1.59 ^c^	<0.001
	Diurnal (%)	28.56 ± 1.35 ^a^	21.06 ± 1.20 ^b^	32.01 ± 1.59 ^a^	<0.001
**Feed rate (mg/s)**	Total	7.87 ± 0.82 ^a^	3.65 ± 0.83 ^b^	2.8 ± 0.31 ^b^	<0.001
	Nocturnal	8.22 ± 1.07 ^a^	3.13 ± 0.38 ^b^	2.84 ± 0.38 ^b^	<0.001
	Diurnal	7.42 ± 0.71 ^a^	3.98 ± 1.41 ^b^	2.44 ± 0.27 ^b^	<0.001

^a^^,b^^,c^ Values in the same row with varying superscripts are significantly different based on Tukey’s *post hoc* comparisons (*p* < 0.05).

**Figure 1:**
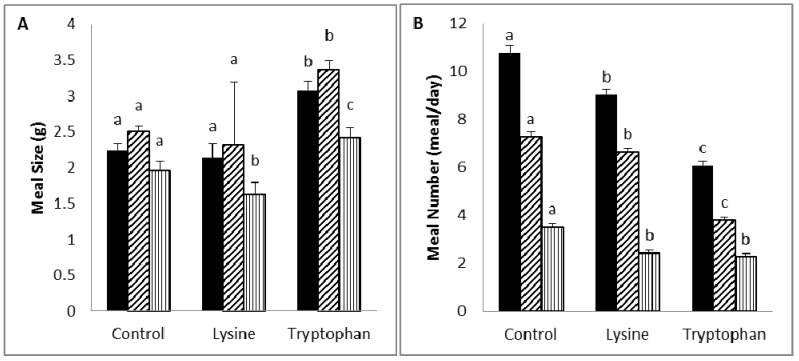
Mean meal size (g) (**A**) and meal number (**B**) of rats fed on diets supplemented with 5% lysine or tryptophan.

The total meal size is the average of the nocturnal and diurnal meals. The total meal number is the total number of meals through an entire day; total (■), nocturnal ( ), diurnal (). ^a^^,b^^,c^ Values in the same bar color with varying superscripts are significantly different based on Tukey’s *post hoc* comparisons (*p* < 0.05).

Meal pattern (Figure 1 and Table 4): The meal size (total, diurnal and nocturnal) of the tryptophan group was significantly higher than those of the other groups. The meal number (total and nocturnal) of the tryptophan group was lower than those of the other groups, and that of the lysine group was lower than the control group. The diurnal meal numbers of the tryptophan and lysine groups were similar, and these were lower than that of the control group.

**Table 4:** Mean meal time (s) and intermeal interval (s) of rats fed on diets supplemented with 5% lysine or tryptophan.

Groups		Control	Lysine	Tryptophan	ANOVA
		(*n* = 10)	(*n* = 10)	(*n* = 10)	*p*-value
**Meal Time (s)**	Total	553.70 ± 124.47 ^a^	1,197.02 ± 65.69 ^b^	2,049.02 ± 146.38 ^c^	<0.001
	Nocturnal	810.45 ± 353.87 ^a^	1,308.53 ± 83.30 ^a^	2,189.22 ± 185.58 ^b^	<0.001
	Diurnal	384.09 ± 23.91 ^a^	766.34 ± 58.23 ^b^	1,636.99 ± 204.26 ^c^	<0.001
**Intermeal Interval (s)**	Total	6,633.46 ± 216.86 ^a^	5,860.19 ± 192.34 ^b^	9,372.29 ± 370.50 ^c^	<0.001
	Nocturnal	5,468.52 ± 215.90 ^a^	5,068.18 ± 156.60 ^a^	7,343.75 ± 348.26 ^b^	<0.001
	Diurnal	10,221.69 ± 676.75 ^a^	5,355.08 ± 673.87 ^b^	11,727.40 ± 992.55 ^a^	<0.001

^a^^,b^^,c^ Values in the same row with varying superscripts are significantly different based on Tukey’s *post hoc* comparisons (*p* < 0.05).

The meal time (total, diurnal, nocturnal) of the tryptophan group was lower than those of the lysine and control groups, and that of the lysine group was lower than that of the control group. The total intermeal interval of the tryptophan group was higher than those of the other groups, while that of the lysine group was lower than that of the control group. The diurnal intermeal interval of the lysine group was lower than those of the other groups, while the nocturnal intermeal intervals of the tryptophan groups were higher than those of the other groups.

### 3.2. Experiment 2

Body Weight and food efficiency (Table 5): The mean initial body weight was similar among the groups; the final body weight was also similar among the groups. The weight gain of the threonine group was significantly lower than those of the control and arginine groups, while the food efficiency was similar among the different groups.

Food intake and feeding rate (Table 6): The total food intake of the arginine group was significantly higher than those of the other groups. The diurnal (%) intake was similar among the different groups, while the nocturnal (%) food intake of the threonine groups was lower than that of the control and arginine groups. The feeding rate (total, diurnal and nocturnal) was similar among the different groups.

**Figure 2:**
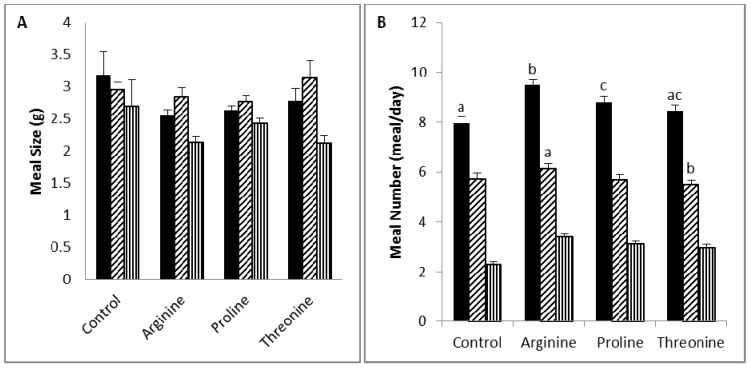
Meal size (**A**) and the number of rats (**B**) fed on diets supplemented with 5% arginine, proline or threonine.

Meal pattern (Table 7 and Figure 2): The meal size (total, diurnal and nocturnal) was similar among the different groups. The total meal number of the arginine group was higher than those of the other groups, and that of the proline group was higher than that of the control group. The diurnal meal number was similar among the different groups. The control and proline groups had similar nocturnal meal numbers, and these were lower than that of the arginine group, but higher than that of the threonine group. Meal time (total, diurnal, nocturnal) was similar among the different groups. The total intermeal interval of the arginine group was lower than those of the control and proline groups. The diurnal meal intermeal interval of the control group was higher than those of the other groups, while the nocturnal intermeal interval was similar among the different groups.

**Table 5:** Mean weight (g) of rats fed on diets supplemented with 5% arginine, proline or threonine.

Groups	Control	Arginine	Proline	Threonine	ANOVA
	(*n* = 6)	(*n* = 8)	(*n* = 8)	(*n* = 8)	*p*-value
Initial body weight (g)	272.14 ± 9.29	284.62 ± 6.64	286.86 ± 7.34	285.94 ± 5.96	NS
Final body weight (g)	385.83 ± 7.16	408.51 ± 11.63	396.63 ± 9.44	375.13 ± 4.08	NS
Weight gain per day (g)	7.58 ± 0.43 ^a^	8.26 ± 0.62 ^a^	7.02 ± 0.34 ^a,b^	5.95 ± 0.38b	<0.01
Food efficiency (weight gain (g)/100 kJ)	1.923 ± 0.165	1.99 ± 0.11	1.81 ± 0.165	1.65 ± 0.11	NS

^a^^,b^ Values in the same row with varying superscripts are significantly different based on Tukey’s *post hoc* comparisons (*p* < 0.05).

**Table 6:** Food intake and feed rate of rats fed on diets supplemented with 5% arginine, proline or threonine.

Groups		Control	Arginine	Proline	Threonine	ANOVA
		(*n* = 6)	(*n* = 8)	(*n* = 8)	(*n* = 8)	*p*-value
**Food Intake**	Total (kJ/day)	383.29 ± 9.10 ^a^	415.32 ± 8.92 ^b^	400.22 ± 7.10 ^a^	376.19 ± 14.74 ^a,c^	<0.05
	Nocturnal (%)	72.89 ± 2.37 ^a^	68.43 ± 1.66 ^a,b^	65.52 ± 1.50 ^a,c^	66.55 ± 1.67 ^c^	<0.001
	Diurnal (%)	27.41 ± 2.88	31.85 ± 1.71	34.34 ± 1.67	33.54 ± 3.06	NS
**Feed rate (mg/s)**	Total	6.78 ± 1.08	5.41 ± 0.46	5.54 ± 0.14	6.93 ± 1.65	NS
	Nocturnal	5.76 ± 0.16	5.53 ± 0.58	5.79 ± 0.25	5.32 ± 0.20	NS
	Diurnal	6.30 ± 1.11	4.99 ± 0.31	5.20 ± 0.13	8.69 ± 3.60	NS

^a^^,b^^,c^ Values in the same row with varying superscripts are significantly different based on Tukey’s *post hoc* comparisons (*p* < 0.05).

**Table 7:** Meal time and intermeal interval of rats fed on diets supplemented with 5% arginine, proline or threonine.

Groups		Control	Arginine	Proline	Threonine	ANOVA
		(*n* = 6)	(*n* = 8)	(*n* = 8)	(*n* = 8)	*p*-value
**Meal Time (s)**	Total	560.29 ± 40.58	571.93 ± 24.44	495.56 ± 12.73	536.36 ± 35.5	NS
	Nocturnal	609.22 ± 63.49	617.81 ± 33.79	505.42 ± 15.67	561.33 ± 37.48	NS
	Diurnal	449.11 ± 32.41	505.99 ± 27.37	485.34 ± 14.86	423.03 ± 20.78	NS
**Intermeal interval (s)**	Total	8,208 ± 344 ^a^	7,289 ± 182 ^b^	8,138 ± 224 ^a^	7,897 ± 277 ^a^^,b^	<0.01
	Nocturnal	6,703 ± 345	6,352 ± 214	6,955 ± 241	7,138 ± 327	NS
	Diurnal	14,083 ± 1168 ^a^	10,451 ± 547 ^b^	11,074 ± 635 ^b^	10,766 ± 777 ^b^	<0.05

^a^^,b^ Values in the same row with varying superscripts are significantly different based on Tukey’s *post hoc* comparisons (*p* < 0.05).

The total meal size is the average of the nocturnal and diurnal meals. The total meal number is the total number of meals through an entire day; total (■), nocturnal (), diurnal (). ^a^^,b^^,c^ Values in the same bar color with varying superscripts are significantly different based on Tukey’s *post hoc* comparisons (*p* < 0.05).

## 4. Discussion

This research attempts to shed light on the physiological processes controlling feeding activity, namely satiation and satiety. Satiation is signposted by the meal duration and/or meal size; *i.e.*, an increase in satiation is reflected in a decrease in meal time and/or meal size. On the other hand, satiety is defined by the time between meals and the number of meals; *i.e.*, a decrease in satiety is indicated by an increase in the number of meals and a reduction in intermeal intervals. As such, studying meal patterns (meal size, meal number, intermeal interval, meal time) provides valuable information on the mechanism by which nutrients may influence feeding activity (satiation or satiety) [8]. In the present study, total food intake refers to all food consumed within 24 h and includes any intake outside of the defined meal. The amount of food consumed outside of the meal was very small, and this is not expected to impact the results.

Large variations (up to 50%) in individual amino acids are present between different proteins, and thus, a 5% addition of amino acid was chosen to mimic the dietary variation of proteins among individuals. Such an amount is not expected to cause adverse effects, since healthy animals receiving adequate quantities of all essential nutrients tolerate a considerable dietary disproportion of amino acids without exhibiting adverse effects [36]. Amino acids were reported to affect taste [37], and both taste and flavor aversions are known to decrease the eating rate. The consistency in the feeding rate among the different groups in Experiment 2 indicates that arginine, proline and threonine supplementation did not result in a significant effect on food palatability. However, lysine and tryptophan seem to have impacted the palatability of the diet.

Diet supplementation with lysine (5%) was associated with a reduction in body weight or weight gain, due to a reduction in food intake and efficiency, which is likely to be the result of an increase in diet-induced thermogenesis. Lysine was reported to be a potent anorectic amino acid in rats, and its anorectic activity may relate to its activity in delaying gastric emptying and inducing neuronal activity at the vagal afferent [31]. In contrast, lysine-deficient diets have been reported to decrease food intake, and this has been shown to be reversed by the addition of lysine [38,39]. Thus, both under and over consumption of lysine seem to reduce food intake. While lysine supplementation of subjects at risk of lysine deficiency was found not to affect body weight, it exerted other beneficial effects [40,41]. In humans, lysine ingestion with glucose has been reported to increase postprandial glucose clearance, while insulin was not altered [26]. This may have been behind the reduction in diurnal meal size, which is known to be reduced by insulin [42]. However, the increased satiety in the lysine group may have been related to the excitatory effect of lysine on the secretion of the gut hormones, CCK and GLP-1 [27], which are known to decrease appetite, mainly through a reduction in meal number. In addition, the sustenance of the meal size may have been the consequence of the stimulation of a compensatory mechanism to maintain food intake [43].

Tryptophan supplementation (5%) caused a reduction in body weight or weight gain through a decrease in both food efficiency and intake, which was the result of an increase in satiety and a decrease in satiation. Similar to lysine, tryptophan was found to induce an anorectic effect in rats, and this is believed to be attributed to its activity in delaying gastric emptying and inducing neuronal activity at the area postrema [31]. This is in contrast to the effect of increased brain serotonin (a byproduct of tryptophan) [44,45], which is known to have a negative effect on appetite [45] by increasing satiation or decreasing meal size [46]. Serotonin was reported to block the effects of the appetite-enhancing neurotransmitter, NPY, in the paraventricular nucleus (PVN) and to have a direct effect on the serotonin receptor in the brain, causing a decrease in food intake and an increase in after-meal satiety (a decrease in meal number) [47]; the latter is in line with our findings.

Food intake and meal pattern are partially related to the interactions between serotonin and dopamine in the brain, and the status of these neurotransmitters depends on their brain uptake and the intake of their precursors (tryptophan, phenylalanine-tyrosine), which are known to compete for uptake by the brain. The interaction at the lateral hypothalamus (LH) has been reported to influence meal size, while the interaction at the ventromedial hypothalamus (VMN) affects meal number [46]. Thus, in our study, an interaction at both LH and VMN may have been present as indicated by the observed alteration in both meal size and number. Increased tryptophan intake is likely to reduce phenylalanine-tyrosine brain uptake and, thus, brain dopamine concentration, and this, in turn, would be expected to decrease the meal number. Since reduced brain dopamine is known to be associated with the inability to initiate feeding, this causes a reduction in meal numbers, leading to an increase in intermeal intervals [48]. However, tyrosine supplementation, a precursor of dopamine, has been shown not to affect meal numbers, and this may have been related to its capacity to induce insulin release [24], which is known to increase meal numbers [42]. In addition, the observed reduction in meal numbers is in line with the excitatory effect of tryptophan on the secretion of the gut hormones, CCK and GLP-1, which are known to reduce meal numbers [27]. However, the observed increase in meal size and time is in contrast to the known effect of the peripheral or central injection of serotonin on meal size [49]. Thus, tryptophan involvement in meal pattern seems to operate beyond its role as a precursor of serotonin. This may partially explain the failure of tryptophan supplementation (of a diet containing 2.5 g tryptophan/kg dry matter) with up to a 1-g tryptophan/kg diet to affect the food intake and growth rate of young pigs [50].

Diet supplementation with arginine (5%) caused a slight, but not statistically significant, increase in weight gain and a significant increase in food intake, mainly due to a decrease in satiety, as indicated by increased meal numbers and decreased intermeal intervals. Arginine is a precursor of nitric oxide and an inducer of growth hormone release [28,29], which increases weight gain [51] and adiposity [52,53] in humans. Moderate intakes of arginine have been reported to have anti-obesity effects in diet-induced obesity rats maintained at moderate doses (0.2% to 1.5% in drinking water) and in humans receiving about 8.3 g/day (~80 mg/kg body weight per day) [54]. On the other hand, the dietary arginine supplementation (0.2% and 0.4%) of milk-fed young pigs was reported to increase body weight and weight gain, while food intake was not affected [29]. Moreover, the arginine supplementation (1%) of growing-finishing pigs increased body weight gain, and this was associated with an increase in skeletal muscle content and a decrease in fat carcass content [55], while our higher dose increased food intake. Thus, the relation between arginine supplementation and body weight and food intake does not follow a linear pattern, in which low intake produces an anti-obesity effect, while high intake stimulates body weight gain and food intake.

Reduced weight gain in the threonine group in the face of normal food efficiency may be explained by the slight reduction in food intake that reached significance in the nocturnal period. Threonine content ranging from 5.5 to 7.8 g/kg of dietary intake was reported to improve the food intake and weight gain of broiler chickens [30], and optimal growth requires a specific lysine to threonine ratio in pigs [56]. However, both of the above experiments utilized quantities lowers than that of our experimental 50-g/kg diet. On the other hand, proline supplementation (5%) had a minimal effect on growth and meal pattern. In line with that, a 90-day maintenance diet supplemented with proline at a dose ranging between 0.625% and 5% was reported not to affect the food intake and body weight of rats [57].

Thus, it can be postulated that increased consumption of cereals, which are known to have low lysine content, would favor increased energy intake usually associated with the development of obesity. This seems to be in line with the observed association between increased refined carbohydrate (mainly cereals) consumption and obesity [58]. On the other hand, the consumption of dairy products, a good source of tryptophan, favors a lower energy intake, leading to a decrease in body weight. This postulation is supported by several research findings [59]. Thus, an increased intake of dairy products in combination with a decrease in the consumption of cereals would be expected to have the potential of reducing energy intake.

## 5. Conclusions

In conclusion, dietary supplementation (5%) of proline and threonine was associated with a minimal alteration in meal pattern. Lysine reduced food intake mainly due to an increase in satiety; in contrast, arginine supplementation increased food intake due to a decrease in satiety. Tryptophan reduced food intake drastically due to a decrease in both satiety and satiation.
